# Natural Inhibitors of Amyloid Aggregation

**DOI:** 10.3390/ijms241713310

**Published:** 2023-08-28

**Authors:** Paolo Tortora, Francesco A. Aprile

**Affiliations:** 1 Department of Biotechnology and Biosciences, University of Milano-Bicocca, 20126 Milano, Italy; 2 Department of Chemistry, Molecular Sciences Research Hub, Imperial College London, London W12 0BZ, UK; 3 Institute of Chemical Biology, Molecular Sciences Research Hub, Imperial College London, London W12 0BZ, UK

Amyloid aggregates are diverse proteinaceous assemblies, including one or more protein species, wherein the molecules interact according to characteristic patterns. Particularly, a major property shared among aggregates, irrespective of their source, is a high content in intermolecular β-sheets, although they also differ for additional but not marginal features. They all display cytotoxic effects, which are accounted for by different mechanisms, mostly aberrant interactions with cell membranes with resulting derangement of calcium homeostasis, oxidative stress, mitochondrial dysfunction, and inflammation. Furthermore, it is now well established that the most toxic species are the initial products of the aggregation process, which occur in the form of small oligomers made up of few protein/peptide molecules, rather than the final products of the aggregation process, i.e., large, stable fibrillar assemblies [1]. The repertoire of amyloid-triggered diseases is extensive and encompasses not only several neurodegenerative disorders, including the most widespread (notably Alzheimer’s and Parkinson’s diseases), but also systemic amyloidoses [1].

Not surprisingly, a considerable effort has been made several decades into developing and discovering therapeutic agents capable of contrasting the onset and progression of such diseases. In this connection, natural compounds have been attracting more and more attention since at least the end of the past century. This is because a growing body of experimental evidence has spotlighted the wealth of beneficial effects they exert. The Special Issue “Natural Inhibitors of Amyloid Aggregation” nicely fits into this context. It includes five contributions: three Original Articles and two Reviews. Although limited in size, the issue touches on the most prominent topics regarding the subject, suggesting that much is still to be discovered regarding novel compounds and their potential therapeutic applications.

The issue focuses on plant polyphenols and herbal drugs. Plant polyphenols have been extensively reviewed by Leri and Coll. [2], whereas two research papers have specifically investigated the therapeutic potential and inherent limitations of two major members of this family, namely hydroxytyrosol [3] and epigallocatechin-3-gallate (EGCG) [4]. Although the term polyphenol is not well defined, it generally includes a variety of natural products divided into four main classes (phenolic acids, flavonoids, stilbenes, and lignans), whose structure consists of one or more aromatic rings carrying one or more hydroxyl groups. Overall, the most widely investigated are oleuropein, curcumin, resveratrol (a stilbene), and the aforementioned flavonoids EGCG and hydroxytyrosol.

Conversely, an in-depth review of herbal drugs destined for Alzheimer’s disease treatment has been provided by Wuli and Coll. [5]. While they all stem from plant sources, these compounds are very heterogeneous in structure, including polyphenols themselves, but many other types of molecules contain, among others, sugar moieties, aromatic and non-aromatic rings, and aliphatic substituents.

When dealing with antiamyloid compounds, it should be first established whether they should be classified as pharmaceuticals (drugs developed through clinical trials for the treatment of specific diseases) or nutraceuticals (natural compounds, mostly endowed with preventive properties against one or more pathological conditions); although, this distinction may be blurred, as documented below.

Specifically, the compounds extracted from herbal sources presented in the relevant review [5], albeit of natural origin, are mostly handled and investigated as potential drugs. In contrast, the polyphenols reviewed in [2] are essentially regarded as nutraceuticals, so their effects are generally analyzed from this perspective, namely by characterizing their healthy effects in the long term, mostly on an epidemiological basis. However, several studies also focused on their molecular and cellular mechanisms.

In any case, when inspecting the available data, it is immediately obvious that, regardless of their chemical nature and classification as amyloid-inhibiting compounds, the ones presented in the issue are generally endowed with a plethora of beneficial effects, well beyond the sole capability of preventing or modulating the aggregation of amyloidogenic proteins/peptides. These effects may include antioxidant, anti-inflammatory, anti-apoptotic, hypoglycemic, and antiatherosclerotic properties, along with the epigenetic regulation of gene expression. On the whole, these properties make it apparent that a full understanding at the molecular and cellular level of the mechanisms responsible for such positive health impacts still demands further, in-depth investigations. This holds true, in particular, for polyphenols, whose effects have been most often investigated on an epidemiological basis [2], thus highlighting their preventive properties on cancer, type-2 diabetes, cardiovascular diseases, and metabolic syndrome. Thus, plausibly, there is, as yet, plenty of room for the discovery of new therapeutic applications.

Unlike polyphenols, herbal drugs are mostly effective as either modulators or inhibitors of γ-secretase activity in Alzheimer’s disease treatment [5]. Nevertheless, they are also capable of exerting additional beneficial effects (in particular, those acting as γ-secretase modulators rather than inhibitors), which also adds to their therapeutic potential. Overall, this relies upon neurotropic, antioxidant, anti-inflammatory, and anti-apoptotic actions, which are implemented through diverse transduction pathways.

In short, what lessons are taught by the present issue and by ensuing related contributions? Basically, they can be summarized in the following points:

(1) As largely documented above, the biological effects and the related therapeutic potential of the molecules presented and investigated are, as a rule, astoundingly diverse, which gives room for plenty of further research. However, particularly noteworthy in this respect is the capability of some compounds to act effectively towards a repertoire of different diseases. For instance, this is the case of the polyphenol hydroxytyrosol, which was initially proven to be effective in preventing insulin amyloid aggregation [3] and subsequently displayed a protective action against both β-amyloid aggregation [6] and doxorubicin-induced apoptosis in cardiomyocytes [7]. Thus, it is to be expected that for many such drugs, there is room for the discovery of new therapeutic applications.
(2) Interestingly, based on the fact that different molecules generally act via different repertoires of therapeutic mechanisms, it has been suggested that the combined administration of multiple drugs may result in synergistic effects [5], a strategy that generally deserves to be explored when searching for new therapeutic approaches.
(3) In this context, bioinformatics holds a promising potential because of developing new therapeutic molecules, as highlighted by the contribution by Muscat and Coll. [8]. Using a combination of ensemble docking and molecular dynamics, they assessed the destabilizing effect of 57 compounds on the S-shaped Aβ42 (the most stable conformer). In this way, they found that the polyphenols oleuropein, curcumin, gossypin, piceatannol, and the related 6-shogaol appreciably affected protein stability, reducing the percentual content of beta sheets. Even more importantly, the authors suggest that a pharmacophore model might be developed based on the compounds’ shared features to rationally design novel, more effective compounds.
(4) Of course, some caveats should be considered when evaluating the therapeutic effects of the compounds dealt with in the issue, primarily hormesis, as well as possible chemical modifications they might undergo after administration. Hormesis is defined as any process in a cell or an organism characterized by a biphasic response to the exposure to increasing amounts of a stressing condition or substance (stressor) [2]. Briefly speaking, besides their beneficial effects, polyphenols can also act as stressors so that a favorable biological response can only occur within a given range of administered doses (the so-called hormetic zone). In contrast, cell damage can occur when this is exceeded. This obvious precautionary principle cannot be disregarded, particularly when long-term administration is required. As far as the effects of chemical modifications are concerned, the contribution by Sternke-Hoffmann and Coll. is of remarkable interest [4]. When assessing the effect of EGCG on α-synuclein amyloid fibril formation, a complex picture emerged, whereby the process was significantly affected by the chemical-physical conditions and, particularly, the oxidized form of EGCG (whose appearance was prevented at slightly acidic pH and favored by a neutral one) was shown to more effectively prevent aggregation. Based on these observations, one should be aware of the impact on the process of both chemical stability of the compounds under investigation and physiological/working conditions.
(5) Concerning molecules that are supposed to be effective as pharmaceuticals, another factor that should be carefully considered is their bioavailability and efficacy in vivo. This issue is related to several others, including the one mentioned in the previous point 4), wherein the effects of EGCG have been discussed. In a more general perspective, the question arises as to how results achieved using a given experimental model may predict clinical trial outcomes. Regarding γ-secretase modulators, investigations were conducted on several models, including rodents, nematodes, and cell cultures [5]. To date, different molecules of herbal origin are candidates for Alzheimer’s disease treatment, although only one (EVP-0015962) will enter a clinical phase II trial. A major constraint is blood–brain barrier penetrance. Hydroxytyrosol is endowed with this capability [9], which identifies it as a promising neuroprotective agent. Additionally noteworthy is the use of induced pluripotent stem cells (iPSC) [5]. They are excellent cellular models as they can be differentiated into various types of neuronal cells from the stem cell stage, and used as a cellular individualized platform starting from a patient’s iPSC.
(6) In conclusion, what are the most promising developments related to and following this Special Issue? In our opinion, two perspectives are worth mentioning. In the first place, given Alzheimer’s disease treatment, nanoformulations are feasible strategies aimed at improving the low bioavailability of apolar drugs, notably curcumin [10]. In the second place, the modulation of gut microbiota via a suitable dietary intake is coming in the foreground as a major line of action to check Alzheimer’s disease progression, with polyphenols and fibers representing the nutritional compounds with the highest potential of counterbalancing the pathophysiological mechanisms of dementia [11]. Thus, despite the complexities and uncertainties one has to cope with when searching for new therapeutic strategies, there seems to be plenty of room for developing innovative approaches.
